# Giant Intrapericardial Myxoma Adjacent to the Left Main Coronary Artery

**DOI:** 10.3389/fonc.2018.00540

**Published:** 2018-11-21

**Authors:** Piotr Nikodem Rudziński, Barbara Lubiszewska, Jacek Różański, Ilona Michałowska, Mariusz Kruk, Cezary Kepka, Karolina Kryczka, Andrzej Kurowski, Wieslawa Grajkowska, Maciej Pronicki, Marcin Demkow

**Affiliations:** ^1^Department of Coronary and Structural Heart Diseases, Institute of Cardiology, Warsaw, Poland; ^2^Department of Cardiosurgery and Transplantology, Institute of Cardiology, Warsaw, Poland; ^3^Department of Radiology, Institute of Cardiology, Warsaw, Poland; ^4^Department of Anesthesiology, Institute of Cardiology, Warsaw, Poland; ^5^Department of Pathology, The Children‘s Memorial Health Institute, Warsaw, Poland

**Keywords:** cardiac tumor, myxoma, intrapericardial tumor, intrapericardial mass, coronaries

## Abstract

A 62-years-old woman was admitted to the hospital because of chronic cough, expectoration of thick mucus, hoarseness and tightness in the precordial area. Computed Tomography (CT) examination revealed the presence of a giant intrapericardial tumor with the dimensions of 80 × 38 × 32 mm. It was located anteriorly and laterally to the left atrium, posteriorly to the pulmonary trunk and the ascending aorta. This hypodense change modeled the left atrium without evidence of invasion. CT coronary angiography and 3-dimensional reconstruction were applied to enable precise planning of cardiac surgery. CT evaluation confirmed that it is possible to remove the tumor without damage to the adjacent left main coronary artery. The patient underwent cardiac surgery with sternotomy and cardiopulmonary bypass. A cohesive, smooth, vascularized tumor pedunculated to the left atrial epicardium was visualized. The location and dimensions corresponded to those determined by CT scan examination. The entire tumor was successfully dissected together with adjacent adipose and fibrous tissue. Histological evaluation revealed the presence of myxoid cells, blood vessels, degenerative changes, and microcalcifications embedded in profuse hyalinized stroma. Those histological features enabled identification of the intrapericardial tumor as a myxoma. Follow-up CT examination did not demonstrate any signs of recurrence of the myxoma. According to our knowledge, a myxoma located inside the pericardial sac has never been described before.

A 62-years-old woman was admitted to the hospital because of chronic cough, expectoration of thick mucus, hoarseness, and tightness in the precordial area. Laryngological examination, resting ECG, chest X-ray, and spirometry did not show any significant abnormalities. Due to the lack of clinical and laboratory markers of active inflammation the diagnosis was expended by 128-slice Contrast-Enhanced Computed Tomography (CT; Siemens, Germany) of the chest. There were no significant changes in the lungs, however, CT (Figure 1) revealed the presence of a giant intrapericardial tumor with the dimensions of 80 × 38 × 32mm. It was located anteriorly and laterally to the left atrium (LA), posteriorly to the pulmonary trunk and the ascending aorta. This hypodense change modeled the LA without evidence of invasion.

**Figure 1:**
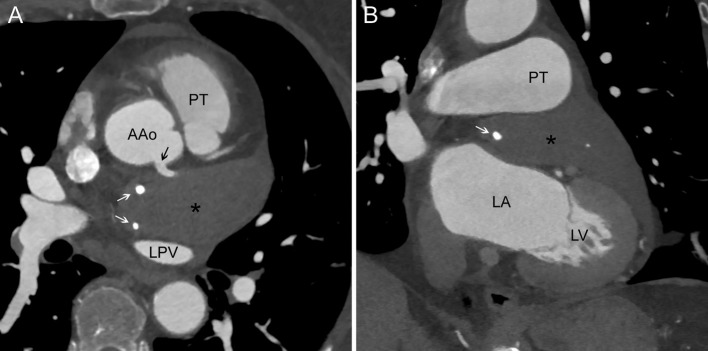
Chest computed tomography. **(A)** Transverse section. The intrapericardial tumor (asterisk) lies between ascending aorta (AAo), pulmonary trunk (PT), and left pulmonary vein (LPV). It contains two macrocalcifications (white arrows) and is modeled by the left main coronary artery (black arrow). **(B)** Coronal section. Left atrium (LA) is slightly compressed by the tumor without any signs of invasion. Left ventricle (LV) models inferior part of the tumor, PT models its superior part.

CT coronary angiography and 3-dimensional reconstruction (Figure 2 and Supplementary Figure 1) were applied to enable precise planning of cardiac surgery. CT evaluation confirmed that it is possible to remove the tumor without damage to the adjacent left main coronary artery. Transthoracic echocardiography confirmed the tumor location; there were no signs of valvular disease, nor heart failure (Supplementary Figure 2).

**Figure 2:**
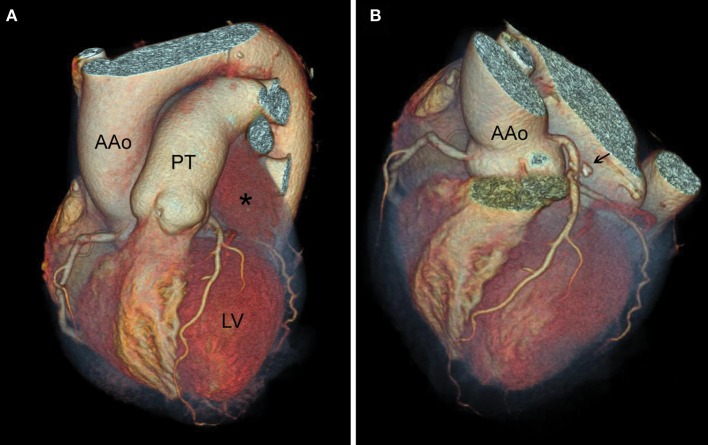
Heart computed tomography, 3-dimensional reconstruction. **(A)** The tumor (asterisk) is located posteriorly and laterally to the pulmonary trunk (PT) and the ascending aorta (AAo), superiorly to the left ventricle (LV). **(B)** Macrocalcificacion (black arrow) as a part of the tumor's structure.

The patient underwent cardiac surgery with sternotomy and cardiopulmonary bypass. A cohesive, smooth, vascularized tumor pedunculated to the left atrial epicardium was visualized (Figure 3A). The location and dimensions corresponded to those determined by CT scan examination. The entire tumor was successfully dissected together with adjacent adipose and fibrous tissue. Histological evaluation (Figures 3B,C and Supplementary Figure 3 revealed the presence of myxoid cells, blood vessels, degenerative changes, microcalcifications embedded in profuse hyalinized stroma, and the absence of papillary structures. Those histological features enabled identification of the intrapericardial tumor as a myxoma. Follow-up CT examination performed after 12 months (Supplementary Figure 4) did not demonstrate any signs of recurrence of the myxoma.

**Figure 3:**
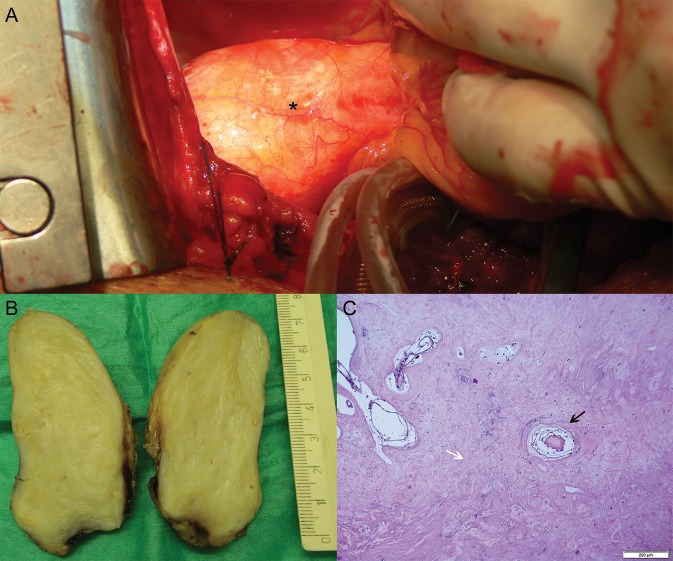
Intraoperative image and histopathology of the tumor. **(A)** Cardiac surgery. Transverse intersection of the aorta and the pulmonary trunk exposed the intrapericardial tumor (asterisk). **(B)** Macroscopic section of bisected mass. The scale indicates its longitudinal dimension of 75 mm. **(C)** Microscopic section. Hematoxylin and eosin-stained histology image of the tumor. A blood vessel surrounded by myxoid cells (black arrow). Microcalcifications and degenerative changes are embedded in profuse hyalinized stroma (white arrow).

The majority of cardiac tumors are secondary metastatic deposits from other sites. Among primary cardiac tumors the largest group is represented by benign lesions, mainly myxomas (1). Some of them present an endocrine function and can be responsible for a number of non-specific symptoms (2). Myxomas are more common in women. Moreover, they may be associated with the presence of chromosomal aberrations. Typically, myxomas are localized in the interatrial septum of the LA. Nevertheless, they may occur within all of the heart cavities and present a local malignant character due to their location, structure, and size (3).

With the aid of CT scanning we were able to outline precisely the anatomy of the intrapericardial tumor and its relations with the heart and non-heart structures. This study may emphasize the significance of CT examination before rare surgical operations. This allows detailed pre-operative planning, aids proper cardiac intervention and reduces the risk of complications. It is extremely useful in the evaluation of anatomically unique cases such as an atypically localized cardiac myxoma that is adjacent to the left main coronary artery. According to our knowledge, a myxoma located inside the pericardial sac has never been described before.

## Author contributions

PR: Manuscript preparation; BL: Patient management; JR: Cardiac surgery; IM: CT analysis; MK: Coronary CT analysis; CK: Coronary CT analysis; KK: Echocardiographic assessment; AK: Peri-operative care and patients management; WG: Histological assessment; MP: Histological assessment; MD: Coordination of the diagnostic and therapeutic process.

### Conflict of interest statement

The authors declare that the research was conducted in the absence of any commercial or financial relationships that could be construed as a potential conflict of interest.

## Supplementary material

Supplementary Figure 1 Coronary arteries computed tomography. **(A**,**B)** Longitudinal section. The left main coronary artery (white arrow) is not affected by the tumor (asterisk). **(C)** Transverse section. Anterior surface of the tumor is modeled by the left main coronary artery with a half of its circumference.

Supplementary Figure 2 Transthoracic echocardiography. There were no significant abnormalities except of the presence of intrapericardial tumor. **(A)** Short axis projection. The tumor (47 × 44 mm) is located between aortic root, distal part of the pulmonary trunk and right pulmonary artery. **(B)** Substernal projection. Tumor dimensions were measured as 75 × 43 mm.

Supplementary Figure 3 Histopathology of the tumor. High magnification microscopic photomicrograph. Hematoxylin and eosin-stained histology image of the myxoma. A blood vessel surrounded by myxoid cells are visible.

Supplementary Figure 4 Chest computed tomography presenting comparative pre- and post-operative scans in the transverse section **(A**,**B)**, as well as, in the coronal section **(C**,**D)**. In the follow-up study carried out after 4 months from the cardiac surgery there were no signs of recurrence of the intrapericardial myxoma (asterisk). The remaining cavity (white arrow) reduced its volume and fulfilled with a connective tissue. AAo indicates ascending aorta; black arrow, left main coronary artery; LA, left atrium; LAA, left atrial appendage; LPV, left pulmonary vein; LV, left ventricle; PT, pulmonary trunk.
