# Supplementary figures and images for: Giant Intrapericardial Myxoma Adjacent to the Left Main Coronary Artery
Source: Front Oncol. 2018 Nov 21;8:540. doi: 10.3389/fonc.2018.00540 (PMC6262358; doi:10.3389/fonc.2018.00540)

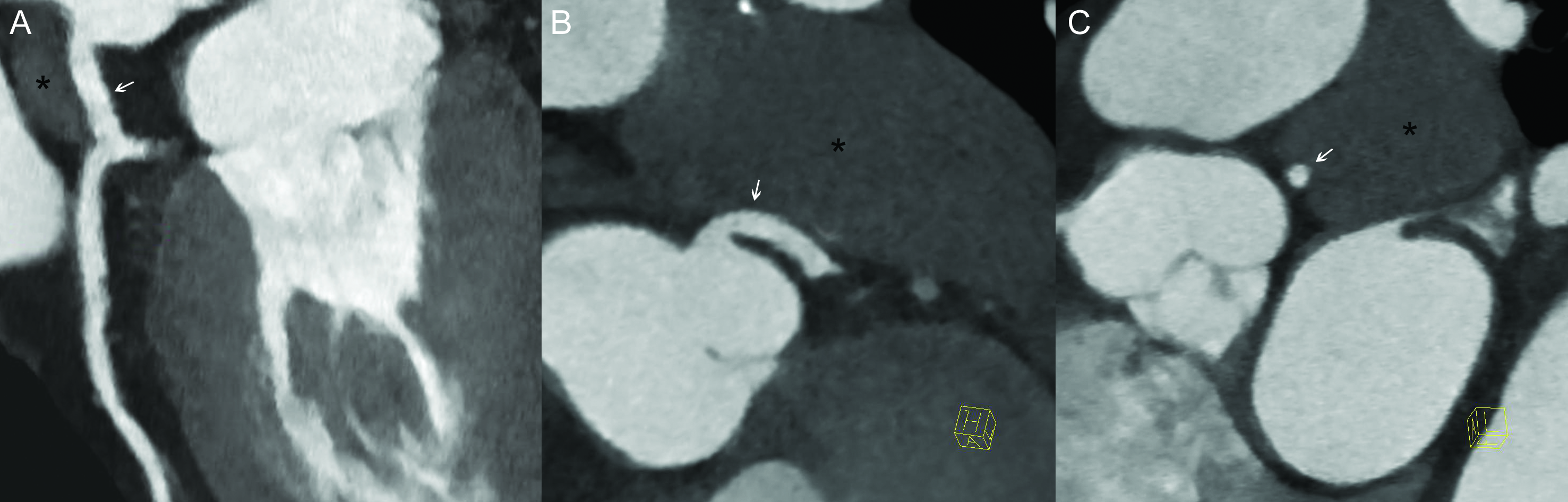

Supplement: Supplementary Figure 1 — Coronary arteries computed tomography. (A,B) Longitudinal section. The left main coronary artery (white arrow) is not affected by the tumor (asterisk). (C) Transverse section. Anterior surface of the tumor is modeled by the left main coronary artery with a half of its circumference. [file Image_1.TIF]

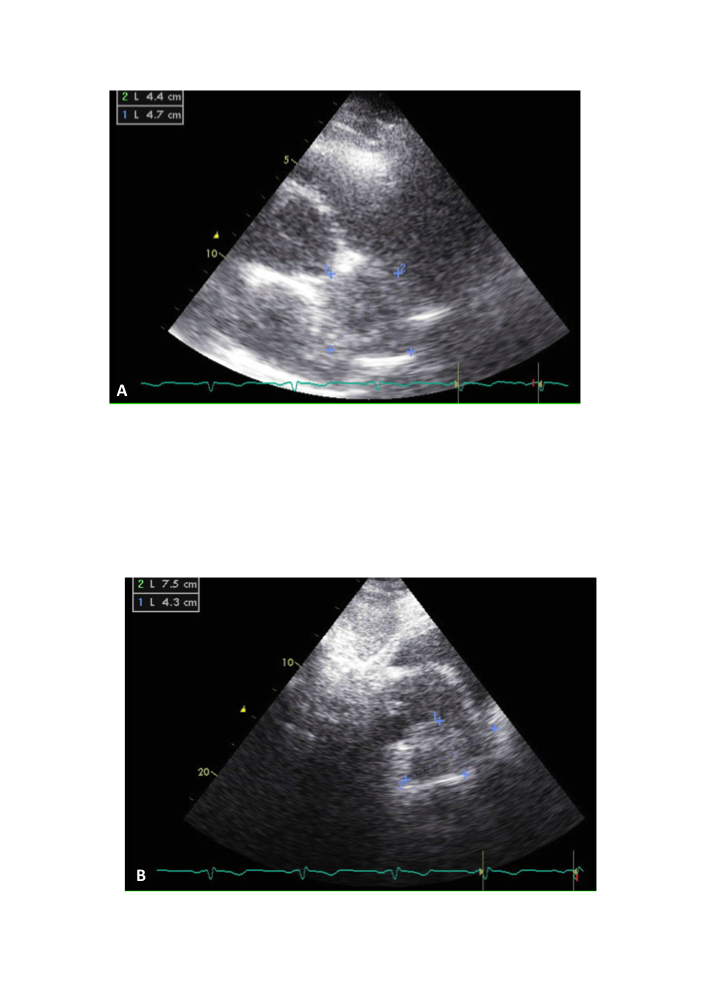

Supplement: Supplementary Figure 2 — Transthoracic echocardiography. There were no significant abnormalities except of the presence of intrapericardial tumor. (A) Short axis projection. The tumor (47 × 44 mm) is located between aortic root, distal part of the pulmonary trunk and right pulmonary artery. (B) Substernal projection. Tumor dimensions were measured as 75 × 43 mm. [file Image_2.tif]

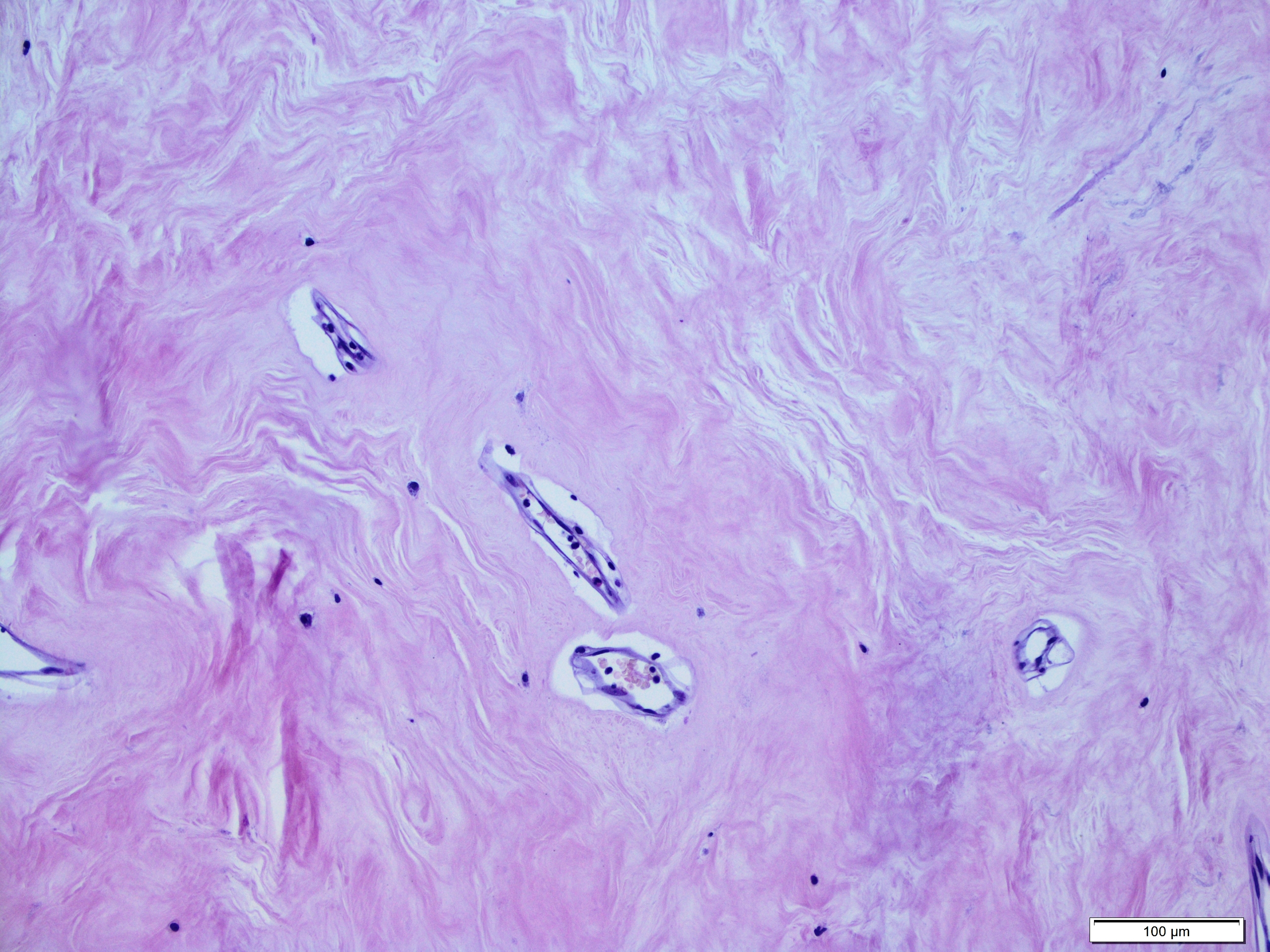

Supplement: Supplementary Figure 3 — Histopathology of the tumor. High magnification microscopic photomicrograph. Hematoxylin and eosin-stained histology image of the myxoma. A blood vessel surrounded by myxoid cells are visible. [file Image_3.jpeg]

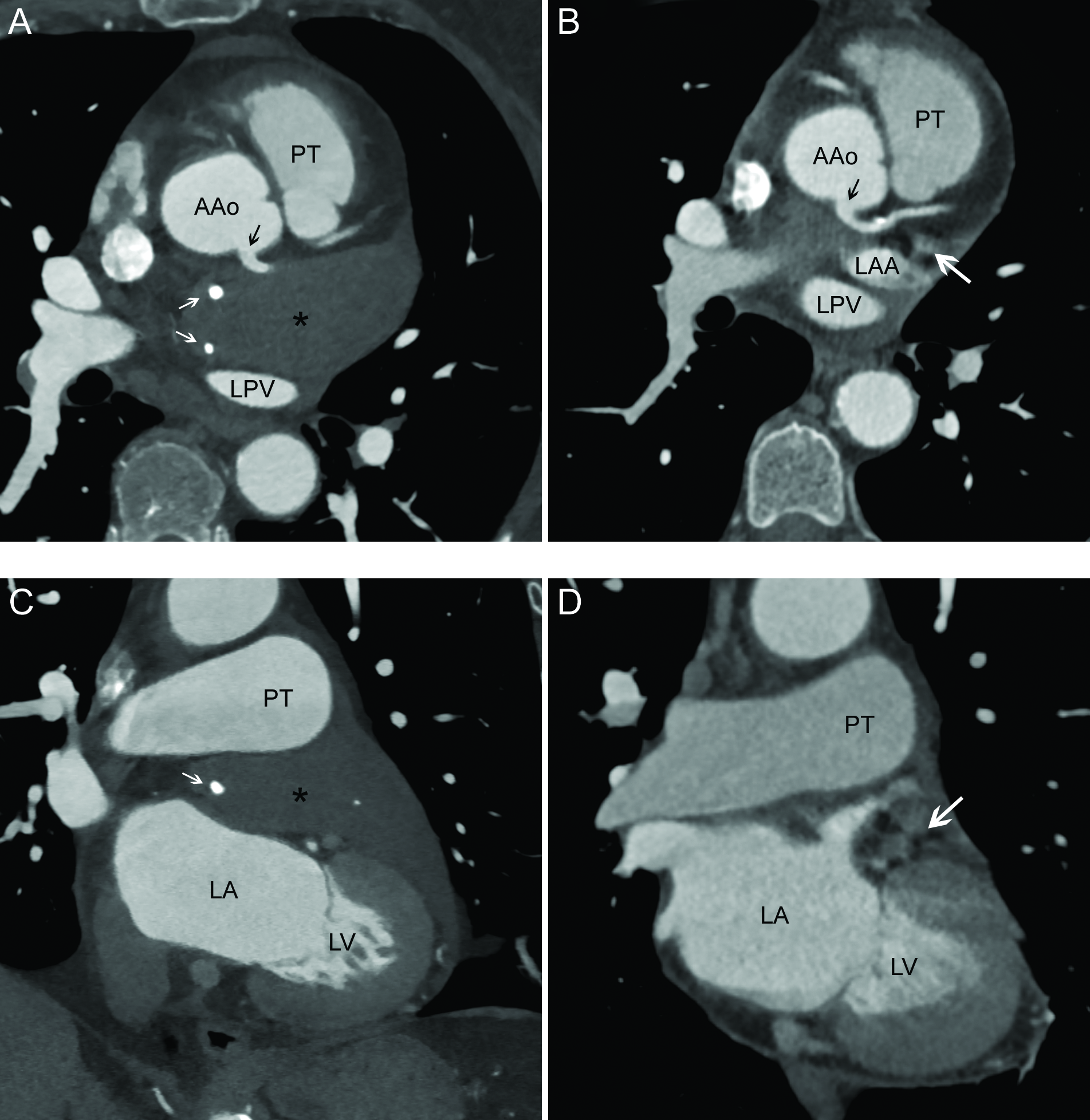

Supplement: Supplementary Figure 4 — Chest computed tomography presenting comparative pre- and post-operative scans in the transverse section (A,B), as well as, in the coronal section (C,D). In the follow-up study carried out after 4 months from the cardiac surgery there were no signs of recurrence of the intrapericardial myxoma (asterisk). The remaining cavity (white arrow) reduced its volume and fulfilled with a connective tissue. AAo indicates ascending aorta; black arrow, left main coronary artery; LA, left atrium; LAA, left atrial appendage; LPV, left pulmonary vein; LV, left ventricle; PT, pulmonary trunk. [file Image_4.tif]
